# Supplementary material for: Association between Traumatic Subarachnoid Hemorrhage and Acute Respiratory Failure in Moderate-to-Severe Traumatic Brain Injury Patients
Source: J Clin Med. 2022 Jul 10;11(14):3995. doi: 10.3390/jcm11143995 (PMC9318973; doi:10.3390/jcm11143995)
Supplement: Supplementary file 1 [file jcm-11-03995-s001.zip › Supplementary material.pdf]

Supplementary files

Supplementary Table S1. Fisher Grading Score

Classification	Description
grade 1	no subarachnoid (SAH)
grade 2	diffuse thin (<1 mm) SAH; no clots
grade 3	localized clots and/or layers of blood >1 mm in thickness; no IVH
grade 4	diffuse or no SAH; ICH or IVH present

The Fisher scale is the initial and best-known system of classifying the amount of subarachnoid hemorrhage on CT scans, and is useful in predicting the occurrence and severity of cerebral vasospasm.

Supplementary Table S2. Basal cistern scale

Classification	Description
grade 1	basal cisterns remain visible
grade 2	basal cisterns compressed or incompletely effaced
grade 3	complete effacement of the basal cisterns

Supplementary Table S3. The variables selected to generate the propensity score.

We selected 21 variables for the propensity-score matched analysis, included age, gender, GCS, comorbidities (hypertension, coronary heart disease, diabetes, chronic obstructive pulmonary diseases, stroke), characteristics of TBI (epidural hemorrhage, subdural hemorrhage, brain contusion, intraventricular hemorrhage, midline shift, degree of

compression of the basal cisterns), medical management (venous infusion>3000ml before ARF, general anesthesia and operation before ARF) and pulmonary events (aspiration, intubation, lung consolidation). The Supplementary Table S3 was a separate file in the zip file.

Supplementary Figure S1. CT scans of different degree of compression of the basal cisterns. Typical pictures of basal cisterns compressed in traumatic brain injury are shown as follow: Basal cisterns remain visible (**grade 1**) (**A**); basal cisterns compressed or incompletely effaced (**grade 2**) (**B**); complete effacement of the basal cisterns (**grade 3**) (**C**).

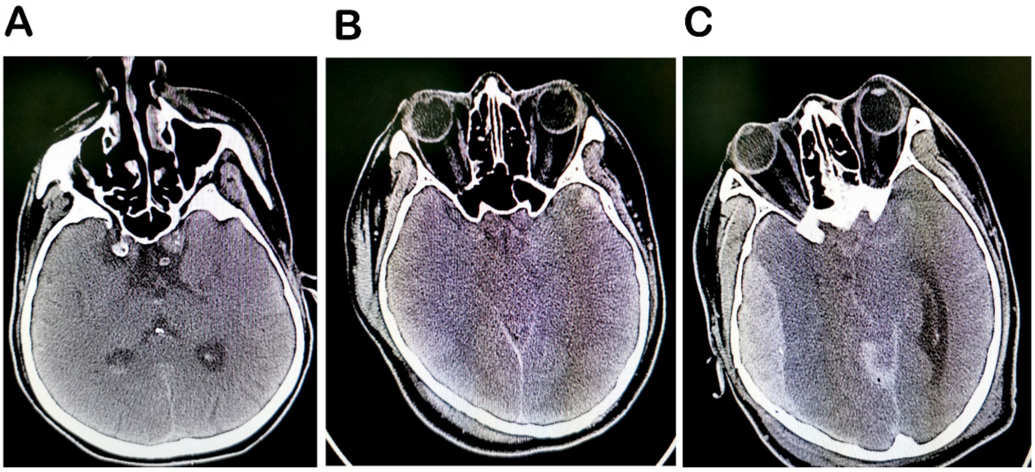

Supplementary Figure S2: The propensity score matching degree was estimated by a standardized mean difference (SMD) between unmatched group and matched group.

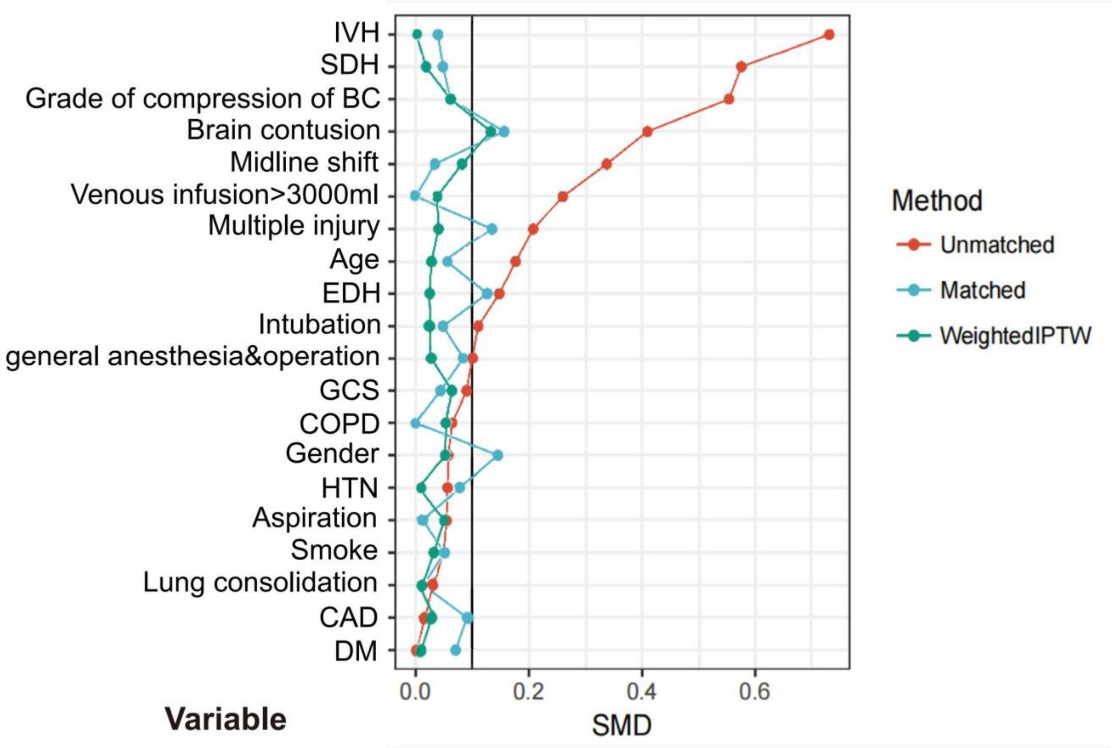

IVH: intraventricular hemorrhage; SDH: subdural hematoma; BC: basal cistern; GCS: Glasgow coma scale; CAD: coronary heart disease; HTN: Hypertension; DM: diabetes mellitus; COPD: Chronic Obstructive Pulmonary Disease; EDH: Epidural hematoma; MLS: midline shift.
